# A similar effect of *P16* hydroxymethylation and true-methylation on the prediction of malignant transformation of oral epithelial dysplasia: observation from a prospective study

**DOI:** 10.1186/s12885-018-4787-6

**Published:** 2018-09-24

**Authors:** Hongwei Liu, Zhaojun Liu, Xue-wei Liu, Si Xu, Lei Wang, Yang Liu, Jing Zhou, Liankun Gu, Yan Gao, Xiao-yong Liu, Huidong Shi, Zheng Sun, Dajun Deng

**Affiliations:** 1 Key Laboratory of Carcinogenesis and Translational Research (Ministry of Education/Beijing), Peking University School of Stomatology, Beijing, 100081 China; 2 Key Laboratory of Carcinogenesis and Translational Research (MOE/Beijing), Division of Aetiology, Peking University Cancer Hospital and Institute, Beijing, 100142 China; 3 Capital Medical University School of Stomatology, Beijing, 100050 China; 4 Georgia Cancer Center, Medical College of Georgia, Augusta University, Augusta, GA 30912 USA

**Keywords:** *P16*, Hydroxymethylation, Oral epithelial dysplasia, Malignant transformation, Prospective cohort

## Abstract

**Background:**

Total *P16* methylation (P16M), including *P16* hydroxymethylation (P16H) and true-P16M, correlates with malignant transformation of oral epithelial dysplasia (OED). Both true-P16M and P16H are early events in carcinogenesis. The aim of this study is to prospectively determine if discrimination of true-P16M from P16H is necessary for prediction of cancer development from OEDs.

**Methods:**

Patients (*n* = 265) with mild or moderate OED were recruited into the double blind two-center cohort. Total-P16M and P16H were analyzed using the 115-bp MethyLight, TET-assisted bisulfite (TAB) methylation-specific PCR (MSP), and TAB-sequencing. Total-P16M-positive and P16H-negative samples were defined as true-P16M-positive. Progression of OEDs was monitored for a minimum 24 months follow-up period.

**Results:**

P16H was detected in 23 of 73 (31.5%) total-P16M-positive OEDs. Follow-up information was obtained from 247 patients with an ultimate compliance rate of 93.2%. OED-derived squamous cell carcinomas were observed in 13.0% (32/247) patients during follow-up (*median*, 41.0 months). The cancer progression rate for total-P16M-positive patients was significantly increased when compared to total-P16M-negative patients [23.3% vs 8.6%; adjusted odds ratio = 2.67 (95% CI: 1.19–5.99)]. However, the cancer progression rates were similar between P16H- and true-P16M-positive OEDs [26.1% (6/23) vs 22.0% (11/50); odds ratio = 0.80 (95% CI: 0.22–2.92)]. The cancer-free survival was also similar for these patients.

**Conclusion:**

P16H and true-P16M are similar biomarkers for determining malignant potential of OEDs. Discrimination of P16H from true-P16M, at least in OED, may be not necessary in clinical applications.

**Trial registration:**

This study is registered prospectively in the U.S. National Institutes of Health Clinical Trials Protocol Registration System (trial number NCT02967120, available at https://ClinicalTrials.gov/ct2/show/NCT02967120).

**Electronic supplementary material:**

The online version of this article (10.1186/s12885-018-4787-6) contains supplementary material, which is available to authorized users.

## Background

Ten-eleven translocation methylcytosine dioxygenases (TET1/2/3) oxidize 5-methylcytosine (5mC) to 5-hydroxymethylcytosine (5hmC), 5-formylcytosine (5fC), and 5-carboxylcytosine (5caC) in the genome [1–4]. Although serial oxidation of 5mC plays a crucial role in active DNA demethylation, a proportion of 5hmC remains stable in the genome without subsequent oxidation and base excision, providing its own regulatory function [5, 6]. In addition to its enrichment in the enhancer and 3′-splice site regions of many genes, 5hmC also exists in CpG islands near the transcription start sites (TSS) of some tumor related genes [7–11]. However, the functions and potential clinical implication of the gene-specific 5hmC content are far from clear.

Traditional bisulfite-based analyses cannot discriminate 5mC from 5hmC [10, 11]. Thus, the result of DNA methylation detection using bisulfite-based methods in fact reflects total methylation, including both true methylation and hydroxymethylation. It is currently unknown whether discrimination of true methylation from hydroxymethylation in commonly used methylation assays will affect the outcome in clinical applications.

Total methylation of the promoter CpG island in the *P16* (*CDKN2A*) gene (P16M) is prevalent in human cancers/precancers [12, 13]. It has been linked to the increased cancer development from epithelial dysplasia in many organs [14–20]. True*-*P16M can directly inactivate gene transcription [21, 22]. We recently reported the presence of dense 5hmC sites in *P16* exon-1 regions in HCT116 cells [23, 24]. Our pilot study showed that *P16* hydroxymethylation (P16H) also occurred in pre-cancer tissues such as oral epithelial dysplasia (OED). In contrast to true methylation of CpG islands around TSS that directly inactivates gene transcription, DNA hydroxymethylation levels are positively correlated with the transcriptional activity of genes in the mammalian genomes [9]. Here, we performed a prospective study to clarify whether the occurrence of hydroxymethylation in the *P16* CpG island affects the predictability of the malignant transformation potential of OED using total-P16M as a biomarker. To the best of our knowledge, we, for the first time, report that detection of P16H is not needed, since the cancer progression rates and progression-free survival are similar between true-P16M-positive OED and P16H-positive OED patients.

## Methods

### Study design

**Fig. 1:**
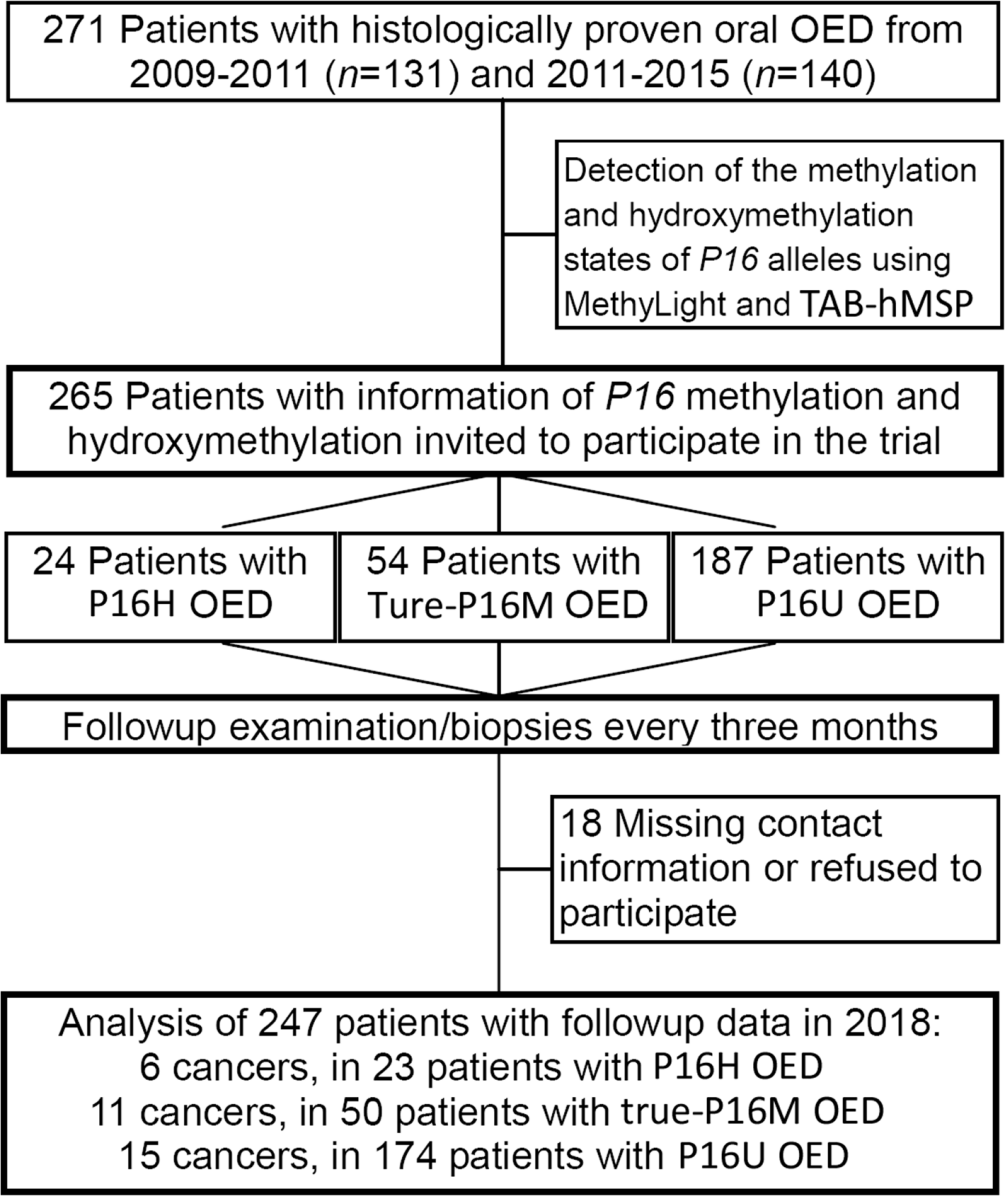
Participant flow diagram. P16H, *P16* hydroxymethylation-positive; True-P16M, true *P16* methylation-positive; P16U, total *P16* methylation-negative

Two hundred sixty-five patients with mild or moderate OED were selected from cases of oral leukoplakia, lichen planus, or discoid lupus erythematosus at Peking University School of Stomatology (Center-A, *n* = 115) and Capital Medical University School of Stomatology (Center-B, *n* = 150). The baseline OED lesions were classified as mild, moderate, or severe by at least two senior pathologists using the same criteria from the 2005 WHO Classification System as we previously reported [19]. Among these OED patients, 128 patients (66 from Center-A and 62 from Center-B) who enrolled in a previously published prospective study [19], started on February 2009 (trial number NCT01695018 at https://ClinicalTrials.gov/ct2/show/NCT02967120; remaining blind for patients and doctors) were included in the present study, because an adequate amount of genomic DNA extracted from OED lesions was still available for the hydroxymethylation analysis. Neither doctors nor patients know the results of *P16* methylation/hydroxymethylation detection during the follow-up period; and laboratory researchers do not know the patients’ follow-up results during *P16* methylation/hydroxymethylation analysis. Therefore, all of these patients were recruited into the double-blind cohort study. Additional 137 OED patients were recruited from 2011 to 2015 (*n* = 49 and 88 from the Center-A and Center-B, respectively) using the same diagnosis and recruitment criteria (Fig. 1).

### Detection of Total-P16M and P16H

To detect total-P16M, genomic DNA was extracted from frozen or formalin-fixed paraffin-embedded (FFPE) tissue samples. The proportion of the methylated copies of a 115-bp fragment in *P16* exon-1 was analyzed using a modified MethyLight assay with bisulfite-modified DNA as the template [25, 26]. Briefly, the sense-strand of the 115-bp methylated fragment in *P16* exon-1 was amplified using forward primer (5’-**CgCg**gt**Cg**tggttagttagt-3′), reverse primer (5’-ta**cG**ct**cG**a**cG**acta**Cg**aaa-3′), and *P16-*specific probe (6FAM-gttgttttt**Cg**t**Cg**t**Cg**gtt-TAMRA). Uracil DNA glycosylase and dUTP were not added into the reaction mixture (Liu et al., 2015). A CpG island-free gene, *COL2A1*, was used as the input reference to prevent false negative detection through monitoring the amount of an input DNA template (the Ct value for *COL2A1* **≤** 29.3 for each included sample) as previously reported [19, 26].

To detect P16H, genomic DNA (3 μg), spiked with both *M.sss*I-methylated and 5hmC-containing λ-DNA controls (Additional file 1: Figure S1), was modified using a TET-Assisted Bisulfite (TAB) Kit according to the manufacturer’s protocol (WiseGene, Cat# K001). During the TAB modification procedures, 5mC was oxidized to 5caC, and then both 5caC and unmethylated-cytosine were subsequently converted to uracil through bisulfite-deamination, whereas 5hmC was protected from oxidation through 5hmC-specific β-glucosylation [10]. The hydroxymethylated *P16* CpG island containing the glucosylated-5hmC was analyzed using TAB-modified templates and a 150-bp hydroxymethylation-specific PCR assay (TAB-hMSP). The primers for P16H were 5’-ttattagagggtggggCggatCgC-3′ (forward) and 5’-GaccccGaaccGcGaccGtaa-3′ (reverse); the primers for non-hydroxymethylated *P16* CpG islands (P16N) were 5’-tTaTTagagggtggggtggaTTgT-3′ (forward) and 5’-cAaccccAaaccAcAaccAtAA-3′ (reverse) [27].

To quantify the proportion of the methylated and hydroxymethylated copies of *P16*, a 392-bp fragment of *P16* exon-1 was amplified using a pair of universal primers (forward, 5′-tttttagaggatttgagggatagg-3′ and reverse, 5′-ctacctaattccaattcccctacaaacttc-3′) with bisulfite- and TAB-modified DNA as templates. The PCR products were analyzed using denatured high performance liquid chromatography (DHPLC) and clone-sequencing [28, 29].

### Definition of true-P16M and P16H status

As previously demonstrated, to consistently detect the fluorescence signal for total-P16M at the proportion of 1/64 (1.56%), each MethyLight reaction (25 μl) should contain at least 8 ng bisulfite-modified template DNA to ensure that each MethyLight reaction produces an informative results [19, 26]. When an amplification signal for the total-P16M is detected in an informative sample, the sample is defined as total-P16M-positive; otherwise, it is considered as total-P16M-negative. Based on the results from TAB-hMSP, the total-P16M-positive samples were further sub-classified into hydroxymethylation-positive (P16H) and true-P16M-positive samples, respectively.

### Follow-up examination and histopathology

The follow-up examination was carried out in a double blind fashion as previously described [19]. If malignant development was observed, an additional examination and re-biopsy were carried out. OED-derived oral squamous cell cancer (OSCC) was histopathologically diagnosed.

### Statistical analysis

Results were displayed by the constituent ratios of enumeration or ranked data. Univariate and multivariate analyses were performed using the Chi Square test and binary logistic regression analysis in SPSS16.0 software. Student’s *t*-test was used to analyze the age data. Patient cancer-free survival was analyzed using the Kaplan-Meier method and log-rank test. Cox’s proportional hazard model was used for multivariate analyses. All *P*-values were two-sided, and *P* <  0.05 was considered statistically significant.

## Results

### Patients’ basic information

Of the baseline mild and moderate OED patients (*n* = 265), 18 cases were lost during the follow-up due to changes to contact information. Thus, 247 cases with follow-up information were ultimately enrolled into the final cohort analysis giving an overall compliance rate of 93.2% (Fig. 1). 73 OEDs were total-P16M-positive; and among them, 23 were P16H-positive. No significant difference in average age, sex ratio, alcohol drinking status, occurrence of Lichen Planus, and lesion grade was observed between total-P16M-positive and -negative OED patients (*P* > 0.05). The proportion of patients with cigarette smoking history was significantly higher in the total-P16M-negative group than the total-P16M-positive group (33.3% vs 17.8%, *P* = 0.014; Table 1). The proportion of patients with tongue OED was significantly lower in the total-P16M-negative group than the total-P16M-positive group (39.1% vs 56.2%, *P* = 0.017).

**Table 1:** Clinical characteristics of patients with oral epithelial dysplasia enrolled into the final follow-up analysis

Status of *P16* methylation and hydroxymethylation	Total-P16M- negative	Total-P16M-positive			Total
Subgroup	(P16U)	(All)	True-P16M	P16H	
Case number	**174**	**73**	**50**	**23**	**247**
Age (yrs, mean±SD)	**55.9±10.3**	**58.1±9.7**	**59.3±9.4**	**55.4±10.1**	**56.6±10.2**
Sex ratio (male, %)	**48.3**	**38.4**	**36.0**	**43.5**	**45.3**
Cigarette smoking (yes, %)	**33.3**	**17.8** ^**a**^	**22.0**	**8.7**	**28.7**
Alcohol drinking (yes, %)	**24.1**	**19.2**	**18.0**	**21.7**	**22.7**
Baseline grade (mild, %)	**69.5**	**58.9**	**58.0**	**60.9**	**59.9**
Lesion site (tongue, %)	**39.1**	**56.2** ^**b**^	**54.0**	**60.9**	**44.1**
Oral Leukoplakia (%)	**69.1**	**30.9**	**21.7**	**9.2**	(*n* = 217)
Oral Lichen Planus (%)	**79.3**	**20.7**	**10.3**	**10.3**	(*n* = 29)
Discoid lupus erythematous	**1**	**0**	**0**	**0**	(*n* = 1)

^a/b^ Fish exact-test, P16U vs total-P16M-positive (All): *P* = 0.014/0.017

### A similar effect of true-P16M and P16H on predicting malignant progression of oral epithelial dysplasia

Malignant transformation of OED to OSCC was observed in 32 of 247 (13.0%) patients during follow-up (range, 14 to 129 months; *median,* 41.0 months). The average baseline age of patients who underwent malignant progression was 3.1 yrs. older than that of patients who remained stable, but not statistically significant (59.3 yrs. vs. 56.2 yrs., *P* = 0.103). The OED lesions of the tongue showed a significantly higher rate of cancer progression than those at other sites (22.0% vs 5.8%, *P* <  0.001).

The progression rate of OED to OSCC in the 73 total-P16M-positive patients was consistently higher than that of the 174 total-P16M-negative (P16U) patients when analyzing different subgroups including: sex, age, baseline grade, lesion site, center/hospital, and specimen storage medium, etc. (Table 2). Multivariate analysis showed that the risk of malignant transformation for total-P16M-positive OEDs was significantly higher than that of total-P16M-negative (P16U) OEDs after adjusting for age, sex, cigarette smoking, alcohol drinking, lesion site, and OED grade [23.3% vs. 8.6%; adjusted odds ratio = 2.67, 95% confidence interval (CI): 1.19–5.99] (Table 2).

**Table 2:** Comparison of malignant transformation of total *P16* methylation-positive and –negative oral epithelial dysplasia in patients with various baseline clinicopathological characteristics

Item		All cases			Total-P16M -negative (P16U)		Total-P16M -positive		Odd ratio (95% CI) in univariate analysis
		*n*	Total-P16M-positive rate (%)	Cancer rate (%)	*n*	Cancer cases (%)	*n*	Cancer cases %)	
Sex									
Male		112	25.0	8.9	84	5 (6.0)	28	5 (17.9)	
Female		135	33.3	16.3	90	10 (11.1)	45	12 (26.7)	2.91 (1.15–7.39)
Age (yrs)									
< 60		153	26.8	11.8	112	9 (8.0)	41	9 (22.0)	3.22 (1.18–8.80)
≥ 60		94	34.0	14.9	62	6 (9.7)	32	8 (25.0)	
Cigarette smoking									
Yes		71	18.3	8.5	58	4 (6.9)	13	2 (15.4)	
No		176	34.1	14.8	116	11 (9.5)	60	15 (25.0)	3.18 (1.36–7.47)
Alcohol drinking									
Yes		56	25.0	8.9	42	2 (4.8)	14	3 (21.4)	
No		191	30.9	14.1	132	13 (9.8)	59	14(23.7)	2.85 (1.24–6.53)
Lesion grade									
Mild		164	26.2	11.6	121	10 (8.3)	43	9 (20.9)	2.94 (1.10–7.82)
Mod.		83	36.1	15.7	53	5 (9.4)	30	8 (26.7)	3.49 (1.02–11.90)
Lesion site									
Tongue		109	37.6	22.0 ^a,c^	68	9 (13.2)	41	15 (36.6)	3.78 (1.47–9.75)
Others		138	23.2	5.8	106	6 (5.7)	32	2 (6.3)	
Center									
A		112	33.0	11.6	75	6 (8.0)	37	7 (18.9)	
B		135	26.7	14.1	99	9 (9.1)	36	10 (27.8)	3.85 (1.41–10.46)
Sample storage									
Frozen		87	32.2	12.6	59	5 (8.5)	28	6 (21.4)	
Paraffin		160	28.1	13.1	115	10 (8.7)	45	11 (24.4)	3.40 (1.33–8.69)
Total		247	29.6	13.0	174	15 (8.6)	73	17 (23.3)	3.22 (1.51–6.87) ^b,d^

^a^ Tongue *vs* Other, *P* < 0.001; ^b^ Total-P16M-negative *vs* Total-P16M-positive, *P* = 0.002; ^c/d^ Adjusted-odds ratio: 0.22 (0.09–0.55)/2.67 (1.19–5.99) , respectively, after sex, age, smoking, alcohol use, lesion site, and lesion grade were adjusted in multivariate analysis. The values are presented in the bold letters when difference between two subgroups is statistically significant.

**Fig. 2:**
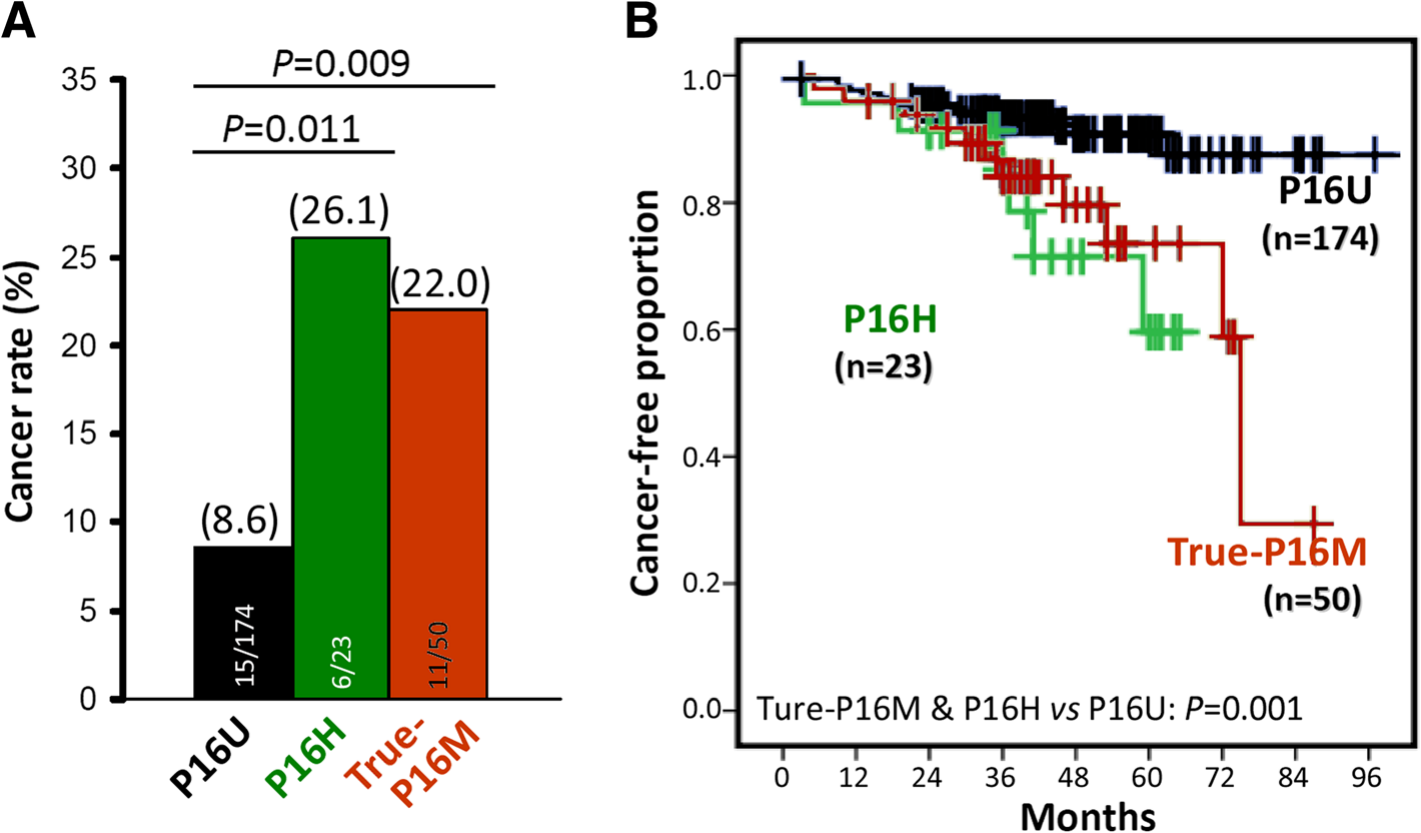
Comparison of the cancer rates of oral epithelial dysplasia (OED) patient groups based on the *P16* alleles with various methylation and hydroxymethylation states. **a** OED-derived cancer rate (%); **b** Estimated cancer-free survival curves in Kaplan-Meier analysis (*P* < 0.001)

Most importantly, among the 73 total-P16M-positive patients, the cancer progression rate between OED patients with and without P16H was not different [26.1% (6/23) vs. 22.0% (11/50); odds ratio = 0.80 (95% CI: 0.22–2.92)] (Fig. 2a). Kaplan-Meier analysis also showed that the cancer-free survival curves were similar between these OED patients with and without P16H, though statistically significant differences were observed between the total*-*P16M-negative (P16U) and total*-*P16M-positive patients (log-rank test, *P* = 0.001; adjusted hazard ratio = 2.21, 95% CI: 1.08–4.54; Fig. 2b).

### Characterization of methylated- and Hydroxymethylated-P16 alleles in OED

**Fig. 3:**
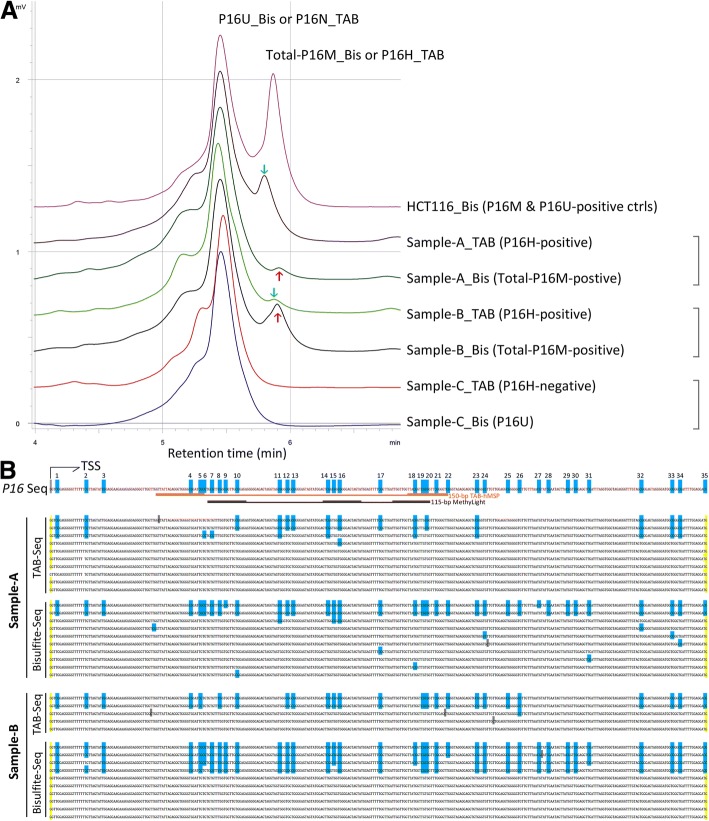
Characterization of the methylation and hydroxymethylation states of CpG sites in a 392-bp fragment of *P16* exon-1 in representative OED samples. **a** DHPLC chromatogram of total-P16M (red arrow-pointed) and total-P16U PCR products amplified from regular bisulfite-templates (Bis); or P16H (green arrow-pointed) and non-hydroxymethylated-*P16* (P16N) PCR products amplified from TAB-templates (TAB). *P16* hemi-methylated cell line HCT116 was used as standard control; Sample-C was total*-*P16M-negative and P16H-negative; **b** Results of TAB-sequencing and bisulfite-sequencing for two representative total-P16M-positive and P16H-positive samples, respectively; each line represents one clone; blue-dot, methylated or hydroxymethylated CpG site; #1–35, CpG ID; location of the 115-bp MethyLight amplicon and the 150-bp TAB-hMSP amplicon were also indicated

The hydroxymethylation and methylation states of the *P16* exon-1 region were further determined using DHPLC in three representative samples. The DHPLC results showed that the retention time for total-P16M was longer than that of P16H for Sample-A and –B (Fig. 3a), suggesting that there were more unconverted CpGs in the bisulfite-treated *P16* exon-1 templates than that of the TAB-treated templates. Clone sequencing further confirmed the DHPLC results. As shown in Fig. 3b, both the bisulfite-sequencing and TAB-sequencing results revealed consecutive unconverted CpGs in some alleles in both Sample-A and –B. Interestingly, CpGs # 27–31 were not hydroxylmethylated in these two samples analyzed. The sequencing results also show that only some alleles are densely methylated, indicate partial methylation in these samples. However, the proportion of alleles with unconverted CpGs was higher in bisulfite-sequencing results than that of TAB-sequencing results, suggesting that the 5hmCs were indeed measured in the bisulfite-sequencing and contributed to the total P16M level.

## Discussions

Numerous studies have demonstrated great potentials of using gene-specific DNA methylation changes as biomarkers for early detection of cancer, diagnosis and prognosis of cancer, and predicting chemotherapy sensitivity and drug resistance. DNA methylation markers may be better suited in clinical oncology practice due to its high sensitivity of detection, high stability in the genome, and relatively low requirements for sample storage [30, 31]. For example, *Sept9* methylation is used as a biomarker for colorectal cancer screening, *MGMT* methylation is used for predicting sensitivity of gliomas to alkylating agents, and DNA methylation panel is used to characterize tissue origin of un-identified cancers [32, 33]. In contract to DNA methylation that stably represses gene transcription, occurrence of 5hmC is positively associated with upregulation of gene transcription [9]. It is likely that DNA hydroxymethylation functions oppositely relative to DNA true methylation in regulation of gene transcription. Therefore, it remains unknown whether occurrence of 5hmC in gene regulatory regions interferences the clinical usages of DNA methylation markers, or whether it is necessary to differentiate DNA true methylation from hydroxymethylation. In the present prospective study we have found, for the first time, that P16H presents in 31.5% (23/73) of total-P16M-positive OED samples and that occurrence of 5hmC in the *P16* CpG islands has no impact on prediction of OED malignant transformation using total-P16M as a biomarker.

Our nested case-control and prospective cohort studies as well as similar studies by others consistently prove that total-P16M is associated with a higher risk of malignant transformation of precancer mucosal epithelial dysplasia in many organs, including oral cavity, esophagus, lung, and stomach [14–20]. The results of present study further confirmed this observation.

We recently found that dense 5hmC sites within methylated-*P16* exon-1 CpG islands in cancer cells may play a role in homeostatic maintenance of methylation of *P16* CpG islands [23]. Unexpectedly, results of the present study did not show detectable difference in OED-derived cancer rate or cancer-free survival between true-P16M-positive OED patients and P16H-positive OED patients. Because the final amount of TAB-template was very limited for most of samples, detection of the P16H proportion in total-P16M-positive samples by MethyLight is not performed. Therefore, it is unknown whether the P16H-positive samples also contain true-P16M. The representative bisulfite- and TAB-sequencing results seem to suggest that 5hmC was only a portion of total 5mC measured by conventional bisulfite-sequencing, though the sequencing results are not very quantitative due to the limited number of clones sequenced.

Our recent study revealed that hydroxymethylated-*P16* alleles in HCT116 cells were transcriptionally inactive [23]. To further study whether hydroxymethylation of the *P16* CpG islands affects gene transcription, we engineered an expression controllable *P16-*specific dioxygenase (P16-TET) and found that DNA demethylation via hydroxymethylation by P16-TET, but not hydroxymethylation itself, could reactivate transcription of methylated *P16* alleles in cancer cells (Gan et al., prepared for publication). These findings suggest that both truly methylated- and hydroxymethylated *P16* alleles are transcriptionally inactive. The results of the present prospective cohort study are consistent with these findings.

*P16* methylation directly inactivates gene transcription and promotes the invasion of cancer cells [21]. Loss of function of *P16* results in higher cyclin D-dependent protein kinase (CDK4/6) activity and thus leads to aberrant phosphorylation of retinoblastoma (RB), which accelerates cell growth. Germline *P16* inactivation by point mutations leads to familial melanoma [34–36]. Both true-P16M and P16H cause *P16* inactivation, this may account for the increase risk of malignant transformation of total-P16M-positive OED lesions.

It was reported that malignant transformation rates for OED patients with and without total-P16M were 27.1% and 8.6% in a double-blind mutiplecentre prospective study [19]. The malignant transformation rates in the present study were very similar to those: 23.3% and 8.6% for OED patients with and without total-P16M. Because 128 OED patients were enrolled in both studies and only 137 additional OED patients were recruited into the two-center study, this may partially account for the similarity.

## Conclusions

**Fig. 4:**
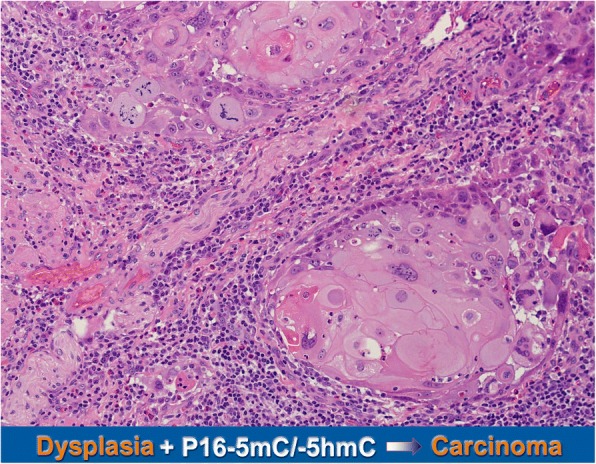
Illustration of both *P16* methylation and hydroxymethylation increase cancer risk of OED

Total-P16M, including true-P16M and P16H, is consistently and significantly linked to malignant transformation of epithelial dysplasia, as previously reported. It is unknown whether or not discrimination of true methylation from hydroxymethylation of *P16* CpG islands is essential in clinical applications. In the present prospective study, we found, for the first time, that cancer risks may not be different between total-P16M-positive OED patients with and without P16H (Fig. 4). Therefore, descrimination of DNA hydroxymethylation from true methylation is not necessary, when total-P16M is used as a biomarker to detect the cancer risk of precancerous lesions.

## Additional file

Additional file 1: **Figure S1.** Characterization of the true methylation and hydroxymethylation states of CpG sites in the *M.sss*I-methylated and 5hmC-containing λ-DNA controls (5mC-Ctrl and 5hmC-Ctrl). Bisulfite-modified DNA templates were used to discriminate 5mC or 5hmC from unmethylated cytosine. TAB-modified DNA templates were used to discriminate 5hmC from 5mC or unmethylated cytosine. The CpG sites within the consensus sequences were listed above the corresponding clone sequences. The number of 5hmC or 5mC sites within each clone was also listed on the left side. These control DNA was added into test samples to monitor the conversion status of 5mC, 5hmC, and unmethylated-cytosine in genomic DNA by bisulfite and TAB treatments. (TIF 197 kb)

## Abbreviations

Bis: Bisulfite modification
DHPLC: Denatured high performance liquid chromatography analysis
hMSP: Hydroxymethylation-specific PCR
OED: Oral epithelial dysplasia
P16H: *P16* hydroxymethylated
P16M: *P16* methylation
P16N: *P16* not hydroxymethylated
P16U: *P16* not methylated
TAB: TET-assisted bisulfite modification
