# Supplementary figures and images for: A similar effect of P16 hydroxymethylation and true-methylation on the prediction of malignant transformation of oral epithelial dysplasia: observation from a prospective study
Source: BMC Cancer. 2018 Sep 24;18:918. doi: 10.1186/s12885-018-4787-6 (PMC6154879; doi:10.1186/s12885-018-4787-6)

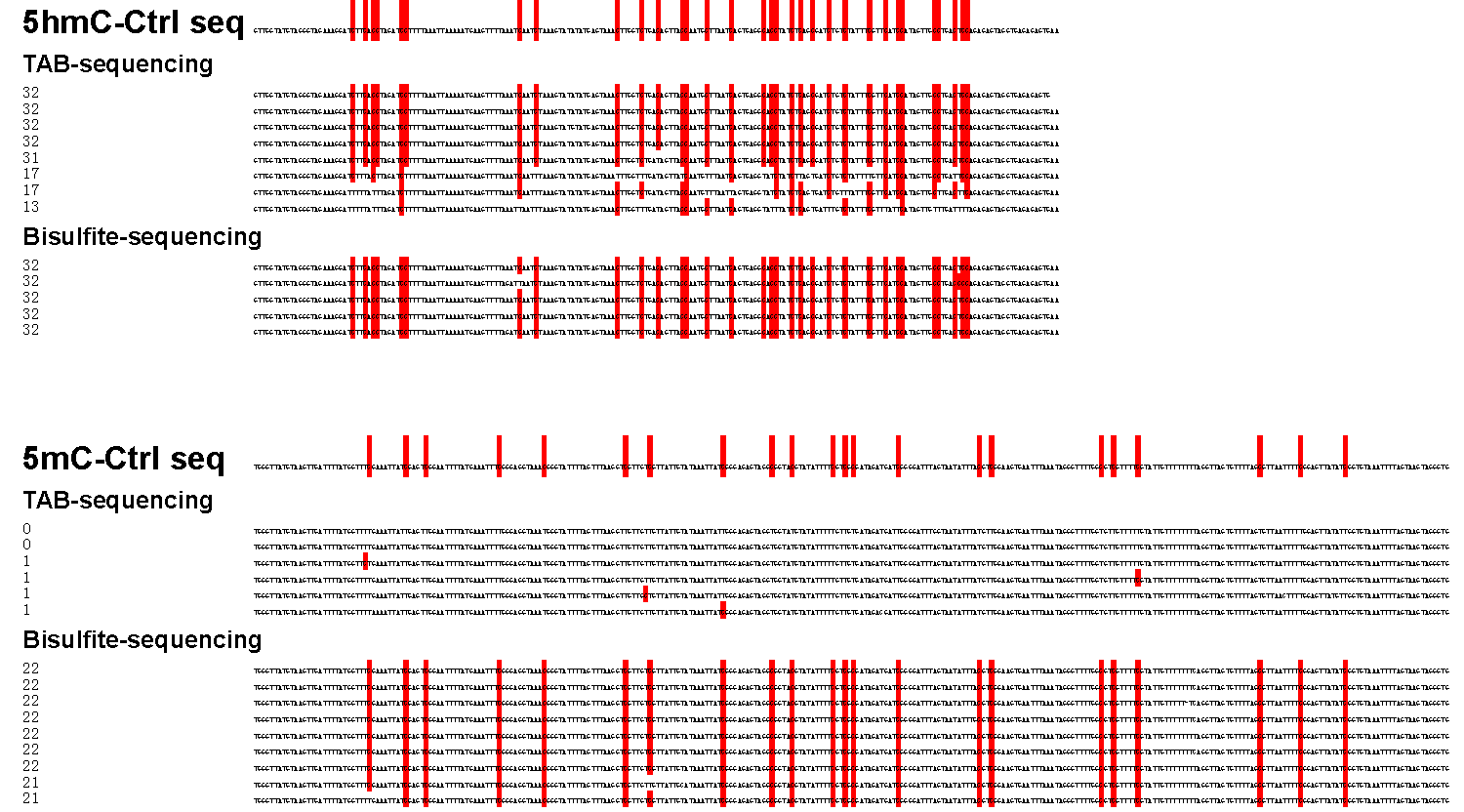

Supplement: Supplementary file 1 — Figure S1. Characterization of the true methylation and hydroxymethylation states of CpG sites in the M.sssI-methylated and 5hmC-containing λ-DNA controls (5mC-Ctrl and 5hmC-Ctrl). Bisulfite-modified DNA templates were used to discriminate 5mC or 5hmC from unmethylated cytosine. TAB-modified DNA templates were used to discriminate 5hmC from 5mC or unmethylated cytosine. The CpG sites within the consensus sequences were listed above the corresponding clone sequences. The number of 5hmC or 5mC sites within each clone was also listed on the left side. These control DNA was added into test samples to monitor the conversion status of 5mC, 5hmC, and unmethylated-cytosine in genomic DNA by bisulfite and TAB treatments. (TIF 197 kb) [file 12885_2018_4787_MOESM1_ESM.tif]
